# Editorial: Basidiomycete fungi: From biosystematics and biodiversity to biotechnology

**DOI:** 10.3389/fmicb.2023.1128319

**Published:** 2023-01-26

**Authors:** Masoomeh Ghobad-Nejhad, Bálint Dima, Bao-Kai Cui, Jing Si

**Affiliations:** ^1^Department of Biotechnology, Iranian Research Organization for Science and Technology (IROST), Tehran, Iran; ^2^Iranian Cryptogamic Herbarium (ICH), Iranian Research Organization for Science and Technology (IROST), Tehran, Iran; ^3^Department of Plant Anatomy, Institute of Biology, Eötvös Loránd University, Budapest, Hungary; ^4^Institute of Microbiology, School of Ecology and Nature Conservation, Beijing Forestry University, Beijing, China

**Keywords:** Basidiomycota, taxonomy, diversity, applied mycology, phylogeny

Basidiomycota is the second-largest phylum of the fungal kingdom and contains a diverse array of fungi. Of the estimated 2.2–3.8 million species of fungi on earth, nearly 150,000 species have been named by now, from which more than 40,000 described species are basidiomycetes (Hawksworth and Lücking, 2017; Antonelli et al., 2020; Bánki et al., 2021; He et al., 2022). They comprise fungi with conspicuous macroscopic fruiting bodies (mushrooms, brackets, coralloids, hydnoids, corticioids, jellys, gasteroids, etc.) to microscopic entities such as rusts, smuts, and yeasts, thriving in an immense range of niches, from terrestrial to aquatic habitats, with a variety of ecological functions, from saprotrophs to parasites, and with diverse kingdom-wide symbiotic associations with plants, lichens, and insects. Basidiomycete fungi play a pivotal role in global edible mushroom production and thanks to their wealth of metabolites, biosynthetic pathways, and survival mechanisms, they have important applications in biotechnology and industry.

Despite their biological diversity and various applications, modern interpretation of several basidiomycete groups is still challenging or they have remained under-documented. Only a few species have been studied in respect of exploitation in biotechnology and the bulk of species have remained untapped. Moreover, the linkage between biosystematics and biotechnology research is still not satisfactory, which would hinder the sustainable utilization of basidiomycete fungi. In support of this endeavor, we proposed the Research Topic “*Basidiomycete fungi: From biosystematics and biodiversity to biotechnology*” with 20 articles from 117 authors from various geographic regions covering various aspects and taxa of basidiomycetes.

Ectomycorrhizae are advanced forms of symbiotic associations of fungi with plants and have a significant impact on the persistence of their host and habitat. Using ITS2 rDNA metabarcoding and rich samplings, Geml et al. studied the effect of abiotic environmental factors on species richness and composition of ectomycorrhizal fungi in the characteristic Pannonian forests in northern Hungary. They showed a particularly strong correlation between ectomycorrhizal composition and pH and soil moisture. Geml et al. concluded that certain topographic or edaphic factors drive the diversity, compositional dynamics, and habitat partitioning of ectomycorrhizal assemblages. Yet, they certified that a large proportion of compositional variance still awaited further exploration.

Ectomycorrhizal basidiomycetes also include some prized edible mushrooms such as *Cantharellus* and *Craterellus* (Hydnaceae, Cantharellales) which are noteworthy for their nutritional properties as well as medicinal values. Six subgenera have been recognized for *Cantharellus*, with the subgenus *Cantharellus* being the largest one (Buyck et al., 2014). Zhang, Lin et al. described four new species of *Cantharellus* subg. *Cantharellus* from China based on morphological evidence and phylogenetic analyses of nLSU and *TEF1* regions. They also discussed the general morphological features, hosts, and species diversity of the subgenus. In another study, Zhang, Zhang et al. studied the genus *Craterellus* in China on the basis of morphological methods and phylogenetic analyses of sequences from the ITS and nLSU regions. They described three new *Craterellus* (*C. fulviceps, C. minor*, and *C. parvopullus*), and provided amended descriptions for two previously known species, with an identification key to the *Craterellus* species known in China.

The genus *Tomentella* (Thelephorales) comprises ectomycorrhizae with thin, resupinate basidiomata and a worldwide distribution. The genus has mainly been reported from temperate and boreal zones of the Northern Hemisphere, with only scarce data from tropical Asia. Lu et al. investigated tropical pine forests in central Vietnam and discovered a high diversity of *Tomentella*. Using molecular phylogenetic analyses of the ITS and nLSU regions and morphological characteristics, six new species (*T. bidoupensis, T. brevisterigmata, T. cinereobrunnea, T. longiechinula, T. stipitobasidia*, and *T. verruculata*) were described and illustrated.

Basidiomycetes also comprise some devastating plant pathogenic fungi causing an economic loss in crop plants and forest trees in different parts of the world. *Tilletia* (Tilletiales, Exobasidiomycetes) is an agriculturally important phytopathogen causing smut in the Poaceae plants (Jayawardena et al., 2019). Currently, *Tilletia horrida*, or kernel smut is considered a noteworthy disease in the US. Khanal et al. provided the first multi-gene analysis to explore the genetic diversity of kernel smut in several cultivated rice populations in the US. Sequences of the ITS, nLSU, *TEF1*, and *RPB1* were analyzed and it was revealed that there were four clades of *T. horrida* populations in the study area. Most of the strains clustered together with 22 *Tilletia* species from different countries in east and west Asia as well as Australia. The results pointed to possible manifold entries of kernel smut into the US and are expected to contribute to the development of effective kernel smut disease management and control.

The genus *Phaeolus* (Polyporales) is a wood-rotting polypore pathogen much known for its type species *Phaeolus schweinitzii*. Yuan et al. made a wide sampling and comprehensive phylogenetic (using ITS and nLSU sequences), morphological, and geographical study of *Ph. schweinitzii*, a common forest pathogen causing butt rot in many commercial timber conifer trees in the Northern Hemisphere. *Ph. schweinitzii* was confirmed to be a species complex including six taxa from three continents. Two new species (*Ph. asiae-orientalis* and *Ph. yunnanensis*) were described from China. The distribution of *Ph. schweinitzii sensu stricto* was shown to be restricted to Eurasia, while the collections originated from North America were concluded to be different species. The genus *Onnia* (Hymenochaetales) is another wood-decaying polypore genus causing tomentose root rot on conifer trees. Zhao et al. studied the phylogeny of *Onnia* based on the ITS, nLSU, *RPB1, RPB2*, and *TEF1* regions and provided a molecular clock analysis which allowed to reconstruct the historical biogeography of the genus. The analyses proposed that the common ancestor of *Onnia* may have emerged in the Paleogene and most species may have evolved in the Neogene. Himalayan region of Asia was suggested as the probable ancestral area of the genus. In addition, the new species *O. himalayana* was discovered in China.

Besides pathogenic wood-inhabiting basidiomycetes, two saprotrophic genera have also been noted in this Research Topic, the brown-rot genus *Fomitopsis*, and the white-rot genus *Panellus*. *Fomitopsis* (Polyporales) inhabits different hardwood and conifer trees. Liu S. et al. investigated the diversity, phylogeny (with nLSU, ITS, mtSSU, nSSU, *TEF1*, and *RPB2* sequences), and ecology of *Fomitopsis*, and described four new species from Sri Lanka, Malasia, and China. They concluded 30 accepted species of *Fomitopsis* worldwide and provided a cumulative map presenting their geographic distribution. Zhang Q. -Y. et al. revised the taxonomy of the poroid members of *Panellus* (Mycenaceae, Agaricales) focusing on the Chinese samples. They described five species new to science (*P. alpinus, P. crassiporus, P. longistipitatus, P. minutissimus*, and *P. palmicola) via* morphological and molecular phylogenetic analyses of five DNA loci (ITS, nLSU, nSSU, mtSSU, and *TEF1*). They also provided an identification key to the 20 known poroid *Panellus* species.

Numerous basidiomycetes are responsible for life-threatening mushroom poisoning around the world. The large genus *Inocybe* and its segregate *Inosperma* (Inocybaceae, Agaricales) are among the prominent poisonous agaric mushrooms. *Inosperma* species are known for neurotoxic poisoning in humans. Li et al. described five new *Inosperma* species from China using morphological characters and phylogenetic analyses of the ITS, nLSU and *RPB2* genes. They also provided an identification key for the 17 Chinese *Inosperma* species. Targeted screening for the important neurotoxins muscarine, muscimol, psilocybin, and ibotenic acid was performed using UPLC-MS/MS. The content of the neurotoxins varied among the new species, while psilocybin was not detected in any. Based on morphological, molecular, and toxin detection evidence, Deng et al. discovered that a poisoning incident occurring in tropical China was caused by a new species described as *Inosperma zonativeliferum*. The UPLC-MS/MS analysis confirmed that the new species contained only muscarine but no other tested toxins. Deng et al. also quantified the muscarine contents in the pileus (2.08 g/kg) and the stipe (6.53 g/kg) of *I. zonativeliferum*.

Gilled mushrooms or agarics (Agaricomycotina) also comprise macrobasidiomycetes with diverse ecological and functional properties. With 558 agaric species in Iran, Ghobad-Nejhad et al. designed a study to unveil the resources of edible, poisonous, and luminescence species in the country. The ecological guilds of the Iranian agarics were also summarized, including the number of ectomycorrhizal, soil saprotrophic, wood-inhabiting, parasitic, and leaf- or litter-inhabiting species. A new approach was applied to showcase the phylogeny of the agaric mushrooms with various antioxidant potentials. It was shown that at least 20% of the species possess antioxidant activity while the edible species would be promising for future functional food developments.

*Gymnopus* sect. *Impudicae* (Agaricales) is well-known for its pungent smell but its diversity is poorly studied. Hu et al. described four new species of sect. *Impudicae* from China using morphology and phylogenetic analysis of nLSU and ITS sequences. They also provided a key to all reported species of *Gymnopus* sect. *Impudicae*. *Micropsalliota* (Agaricales) is an agaric genus containing 62 species with relatively small basidiomata. Yan et al. described six new species of *Micropsalliota* (namely *M. minor, M. ovalispora, M. pseudodelicatula, M. rufosquarrosa, M. tenuipes*, and *M. wuyishanensis*) based on morphological evidence and phylogenetic analyses of ITS, nLSU, and *RPB2* sequence datasets. In addition, an identification key for the 20 Chinese species of *Micropsalliota* was given.

Heterobasidiomycetes comprise basal clades in the basidiomycete phylogeny and include macroscopic and microscopic forms. The corticioid heterobasidiomycetes are comparatively little sampled due to their generally inconspicuous basidiomata. The order Auriculariales is noteworthy as it is home to some of the world-known favorable edible and medicinal fungi. However, due to the overlapping morphological characters, the boundaries of the genera in Auriculariales have remained perplexing. Liu S. -L. et al. revised the generic delimitation in Auriculariales based on phylogenetic analyses of the ITS and nLSU regions and thorough morphological examinations. They introduced the new genus *Alloexidiopsis* belonging to the family Auriculariaceae, and proposed a key to identify its five species. They also summarized the main morphological differences between the nine genera of Auriculariales with corticioid basidiomata, including *Adustochaete, Alloexidiopsis, Amphistereum, Crystallodon, Exidiopsis* (*Heterochaete*), *Heteroradulum, Metulochaete, Proterochaete*, and *Sclerotrema*.

Applied mycology and fungal biotechnology have been fueled by microscopic and yeast ascomycetes on their onset but have soon progressed by studies on several basidiomycetous species. *Auricularia* (ear mushroom) is an economically important fungus with diverse medicinal and nutritional properties, accounting for nearly 17% of the world commercial mushroom production (Royse et al., 2017; Bandara et al., 2019). Phithakrotchanakoon et al. studied the interactions between the cultivated *Auricularia cornea* and the microbiota present in the spent mushroom substrate, applying high-throughput sequencing as well as proteomics analysis of high-yield and low-yield *A. cornea* groups. They showed a significantly higher species richness and diversity of microbiota in the samples of the high-yield group. Moreover, certain bacterial species exhibited high differential abundance between the two groups. Functional analysis of the detected proteins in the bacterial-fungal co-culture showed that elevated mycelial growth of *A. cornea* could be attributed to the concerted actions of four types of proteins. Phithakrotchanakoon et al. concluded that certain bacteria in the substrate foster the growth and yield of *A. cornea* and proposed their results to be utilized for the selection and co-cultivation of growth-promoting bacteria to boost the production yield of *A. cornea*. Their results also provide novel insights into interactions of mycelia with bacteria during the development of the fruiting body.

Fruiting body development in the basidiomycetes has also been subject to the study by Tao et al. who investigated the involvement of a putative GTP cyclohydrolase I (named CaGCH1) in hyphal branching and fruiting body formation in *Cyclocybe aegerita* (Tubariaceae, Agaricales), an edible mushroom cultivated in Asia. Guanosine triphosphate (GTP) cyclohydrolase 1, whose disruption may cause conditional lethality in microorganisms, is the limiting enzyme of the tetrahydrobiopterin (BH4) synthesis pathway. Tao et al. verified that the GCH1 gene was downregulated by constructing an RNAi system for GCH1 in *C. aegerita*.

Growth at low temperatures is a limiting factor for the cultivation of edible and medicinal mushrooms. Ling et al. applied a homologous recombination approach to construct a heat-resistant strain of shiitake (*Lentinula edodes*), the globally second most popular edible mushroom, by the overexpression of the pEHg-gdp-hsp20 vector for the gene hsp20, encoding the heat shock protein 20 in *A. bisporus*. As a result, the overexpression of hsp20 efficiently improved the ability of low-temperature shiitake isolates to defend against heat shock. Their results facilitate the breeding of strains of *L. edodes* with superior transgenic properties.

In another study on *Lentinula edodes*, Kim et al. performed a comparative analysis of the structural properties of the mitochondrial DNAs from 25 strains of this mushroom, 21 of which were obtained *de novo*. While the length of mtDNAs was shown to range from 117 to 122 kb, they had a relatively consistent gene number, with the length variation attributed to the number of introns, repeated sequences, transposable elements, and plasmid-related sequences. Interestingly, the length of *COX1* gene, the said barcode region in some past studies, ranged from 8.4 up to 12 kb. The results showed that mitochondrial DNA is a relatively fast-evolving molecule with insertion, deletion, and repetition events occurring at the strain level.

“Sanghuang” is a common name applied to a group of prized medicinal polypores (*Sanghuangporus* s.l., Hymenochaetales) best known in East Asia. Numerous investigations have revealed that polysaccharides are one of the major bioactive ingredients of sanghuang, with various medicinal properties. Wang et al. comprehensively reviewed the polysaccharides of sanghuang and summarized the recent reports on preparation strategies, structural features, new techniques of structural characterization, bioactivities, and structure-activity relationships. Their study provides valuable guidance for future research on applications of sanghuang polysaccharides.

## Author contributions

All authors listed have made a substantial, direct, and intellectual contribution to the work and approved it for publication.
